# White‐tailed deer consumption of emergent macrophytes mediates aquatic‐to‐terrestrial nutrient flows

**DOI:** 10.1002/ece3.9257

**Published:** 2022-09-12

**Authors:** Jonathan W. Lopez, Daniel C. Allen, Caryn C. Vaughn

**Affiliations:** ^1^ Department of Biology University of Oklahoma Norman Oklahoma USA; ^2^ Oklahoma Biological Survey University of Oklahoma Norman Oklahoma USA; ^3^ Present address: Department of Biological Sciences University of Alabama Tuscaloosa Alabama USA; ^4^ Present address: Department of Ecosystem Science and Management Pennsylvania State University University Park Pennsylvania USA

**Keywords:** aquatic plant, aquatic–terrestrial links, cervid, freshwater, freshwater mussel, macrophyte, stream

## Abstract

1. Trophic interactions between mobile animals and their food sources often vector resource flows across ecosystem boundaries. However, the quality and quantity of such ecological subsidies may be altered by indirect interactions between seemingly unconnected taxa. We studied whether emergent macrophytes growing at the aquatic–terrestrial interface facilitate multi‐step aquatic‐to‐terrestrial resource flows between streams and terrestrial herbivores. We also explored whether aquatic animal aggregations indirectly promote such resource flows by creating biogeochemical hotspots of nutrient cycling and availability.
2. We tested whether white‐tailed deer (*Odocoileus virginianus*) in eastern North America vector nutrient fluxes from streams to terrestrial ecosystems by consuming emergent macrophytes (*Justicia americana*) using isotope and nutrient analyses of fecal samples and motion‐sensing cameras. We also tested whether mussel‐generated biogeochemical hotspots might promote such fluxes by surveying the density and nutrient stoichiometry of *J. americana* beds growing in association with variable densities of freshwater mussels (Bivalvia: Unionoida).
3. Fecal samples from riparian deer had 3% lower C:N and 20% lower C:P ratios than those in upland habitats. C and N isotopes suggested riparian deer ate both terrestrial and aquatic (*J. americana*) vegetation, whereas upland deer ate more terrestrial foods. Motion‐sensing cameras showed deer eating *J. americana* more than twice as frequently at mussel‐generated hotspots than non‐mussel sites. However, mussels were not associated with variation in *J. americana* growth or N and P content—although N isotopes in *J. americana* leaves did suggest assimilation of animal‐derived nutrients.
4. Our findings suggest that white‐tailed deer may conduct significant transfers of aquatic‐derived nutrients into terrestrial habitats when they feed on macrophytes and defecate on land. Whether aquatic animal aggregations promote such resource flows by creating biogeochemical hotspots remains unresolved, but the nearly global distributions of the deer family (Cervidae) and of macrophytes suggest that cervid‐driven aquatic‐to‐terrestrial nutrient flows may be widespread and ecologically important.

## Graphical abstract

Mobile animals often conduct resource flows across ecosystem boundaries. Despite their broad geographic distribution and anecdotal accounts of feeding on aquatic vegetation, white‐tailed deer have yet to be explored as potential vectors for aquatic‐to‐terrestrial resource flows. Here, we found that white‐tailed deer likely mediate transfers of aquatic‐derived nutrients into terrestrial habitats when they feed on macrophytes.

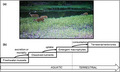

## 1 INTRODUCTION

Ecosystem structure and function constantly respond to exchanges of resources across ecosystem boundaries, known as ecological subsidies. Animals play important roles in conveying resource subsidies in all ecosystem types (McNaughton et al., 1988; Nakano & Murakami, 2001; Polis & Hurd, 1996). Mobile animals may feed in one ecosystem and subsidize another ecosystem via either waste production or mortality. Such subsidies tend to be especially important when conducted across uphill gradients and against the force of gravity. Resource subsidies from aquatic to terrestrial ecosystems tend to traverse this counter‐elevational gradient, with runoff and nutrients collecting at low points in the landscape, making aquatic ecosystems richer in nutrients than terrestrial ones (Schindler & Smits, 2017; Shurin et al., 2006), albeit with exceptions such as high‐elevation lakes or streams that may flood and transfer resources to downhill terrestrial systems. For example, seabirds subsidize coastal ecosystems by excreting marine‐derived nutrients around their nests, thus supporting greater levels of biological production (Polis & Hurd, 1996). The emergence of insects from aquatic larval forms in freshwater habitats to flying adult forms subsidizes terrestrial food webs with energy and nutrients and alters predator–prey dynamics (Baxter et al., 2005; Sabo & Power, 2002). Such direct predator–prey interactions represent some of the best‐known aquatic‐to‐terrestrial resource subsidies. However, multi‐step subsidies created by indirect effects between organisms remain much less explored.

Aquatic vascular plants, or macrophytes, may be especially suited to facilitating multi‐step resource transfers between aquatic and terrestrial animals. Macrophytes are globally distributed and taxonomically diverse, but all grow at the aquatic–terrestrial interface and experience varying levels of submergence beneath—or emergence above—the water's surface (Chambers et al., 2008). Historically, macrophytes were viewed as unimportant to the food web, but a wide range of vertebrate and invertebrate herbivores consume macrophyte tissue (Bakker, Wood, et al., 2016; Lodge, 1991; Newman, 1991). Many macrophyte herbivores are strictly aquatic in nature, such as crayfish, manatees, or fish, but the specific preference that macrophytes exhibit for habitat at the aquatic–terrestrial interface also facilitates exploitation by terrestrial animals—namely insects and large ungulates (Bakker, Pagès, et al., 2016; Newman, 1991). Perhaps the best‐known ungulate herbivore of macrophytes is the moose (*Alces alces*), which enters boreal lakes and ponds to feed on aquatic plant matter. When they return to land, they transfer large amounts of aquatic‐derived nutrients to terrestrial ecosystems (Bump, 2018; Bump et al., 2009). Other species in the deer family (Cervidae) also feed on aquatic macrophytes, but published accounts of this behavior are few and often anecdotal (e.g., Lopez et al., 2020; Takafumi et al., 2015). Authors typically attribute herbivory by cervids on macrophytes to the relatively high concentrations of macronutrients—nitrogen (N) or phosphorus (P)—and essential micronutrients such as sodium, calcium, iodine, or trace metals that they contain (Ceacero et al., 2014; Fraser et al., 1984; Labisky et al., 2003; Watkins & Ullrey, 1983). These anecdotal accounts may be instances of previously unstudied aquatic‐to‐terrestrial resource flows from aquatic sources, through macrophytes at the aquatic–terrestrial interface, and into the terrestrial habitat via deer. Furthermore, aquatic vegetation tends to differ naturally in its isotopic composition compared with terrestrial vegetation—generally more enriched in ^13^C (carbon) and more variable in ^15^N (Finlay & Kendall, 2007; France, 1995). By comparing food plant isotopic signatures with the signatures of feces from cervid herbivores, relative comparisons of the contributions of aquatic and terrestrial food items to a given herbivore's diet can be drawn (Milligan & Humphries, 2010).

**FIGURE 1:**
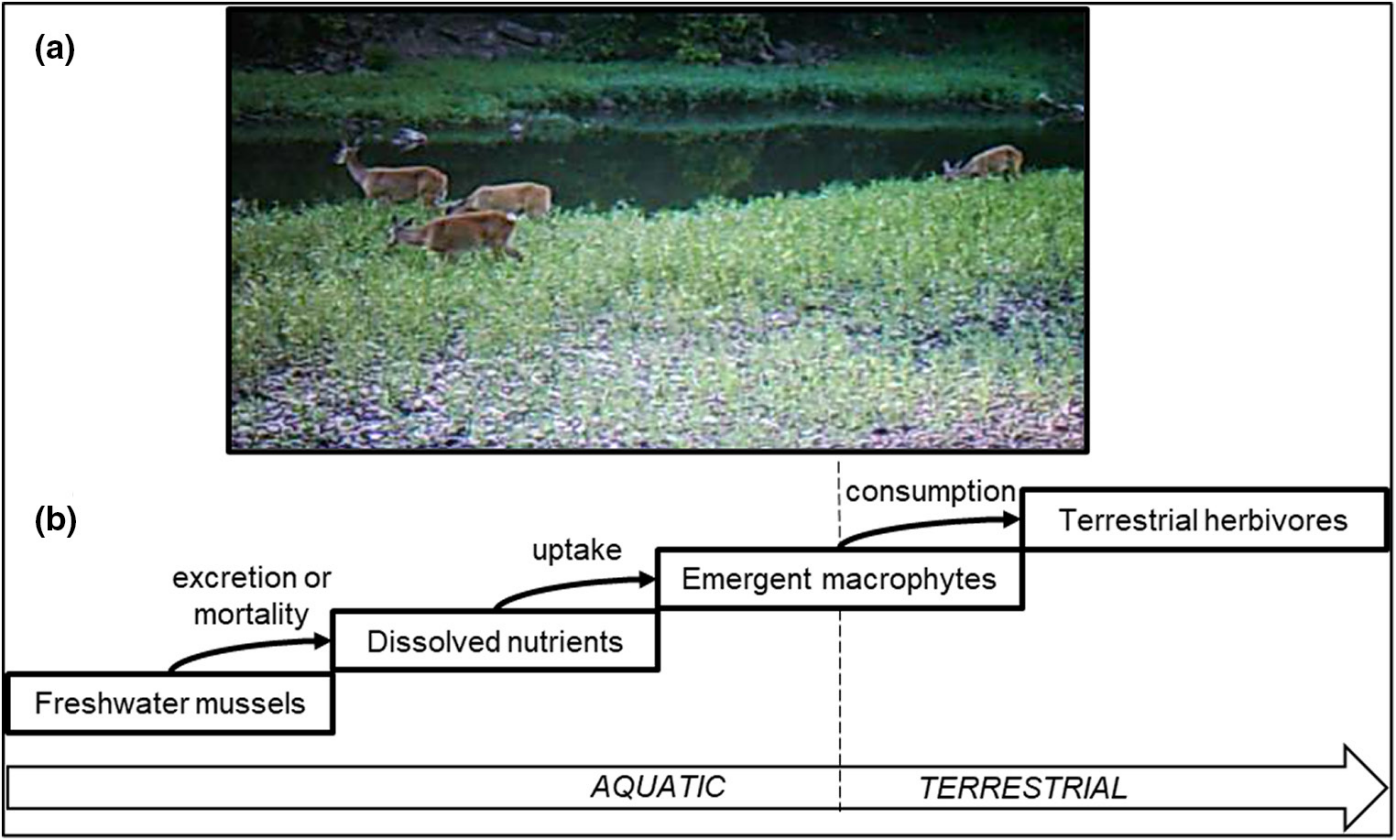
(a) White‐tailed deer (*Odocoileus virginianus*) feeding on emergent macrophytes (*Justicia americana*) on a gravel bar bordering the Kiamichi River, Oklahoma, USA. (b) Conceptual diagram of the hypothesized pathway along which biogeochemical hotspots generated by freshwater mussels may promote indirect aquatic‐to‐terrestrial nutrient subsidies.

In eastern North America, the emergent macrophyte *Justicia americana* is a recently documented food source for white‐tailed deer (*Odocoileus virginianus*) (Figure 1a; Lopez et al., 2020). *J. americana* also has a mutually beneficial relationship with freshwater mussels (Bivalvia: Unionoida, hereafter “mussels”)—the macrophytes improve mussel habitat by stabilizing sediments, while mussel excretion helps meet the plant's macronutrient demands (Atkinson, Kelly, & Vaughn, 2014; Fritz et al., 2004). Mussels create hotspots of N and P cycling in river ecosystems through their filter‐feeding and the resultant excreta (Atkinson & Vaughn, 2015). Increases in mussel density are also associated with increased micronutrient content in *J. americana*, namely calcium and trace metals, which may be due to mussel mortality and the resultant buildup of mussel shell fragments in the ecosystem (Lopez, 2022). Experimental work suggests that the biogeochemical hotspots generated by mussels may also increase macrophyte growth and macronutrient content (Crane et al., 2020; Lopez et al., 2020)—a pattern already identified in marine bivalves (Aquilino et al., 2009; Peterson & Heck, 2001). Because cervids are thought to maximize their nutrient and mineral intake through selective feeding (Fraser et al., 1984; McArthur et al., 1993; Vangilder et al., 1982), mussel‐related effects on macrophyte nutrient content and growth could lead to preferential herbivory by terrestrial consumers of *J. americana* like white‐tailed deer. Such preferential feeding may in turn promote cervid‐driven aquatic‐to‐terrestrial subsidies (Figure 1b).

Here, we explored the possibility that *J. americana* facilitates an indirect pathway allowing white‐tailed deer to transfer aquatic animal‐derived nutrients into terrestrial ecosystems and that freshwater mussel‐generated hotspots may enhance the magnitude and nutritional quality of this subsidy (Figure 1). We conducted a field study in the USA Southern Interior Highlands using stable isotopes (δ^13^C and δ^15^N) and macronutrient stoichiometry (C:N:P) from *J. americana* tissue and from deer fecal pellets in contrasting habitats to determine whether deer consume significant amounts of *J. americana* and whether such consumption increased deer diet quality. We also used motion‐sensing cameras to evaluate whether deer feed more frequently on *J. americana* at mussel‐generated hotspots and used the density and C:N:P stoichiometry of *J. americana* as metrics of the quantity and quality of this macrophyte as a food source. We tested the following hypotheses: (H1) White‐tailed deer fecal samples collected from riparian zones would have relatively higher N and P content and be more enriched in ^15^N and ^13^C compared with those collected from upland ridges bounding the watershed because of access to nutritionally and isotopically enriched macrophytes; (H2) deer more frequently consume *J. americana* from mussel sites compared with other stream segments and terrestrial vegetation because of greater nutrient content; (H3) mussel‐generated hotspots increase ambient N and P concentrations via excretion or mortality, which increases *J. americana* density and/or the relative N and P content of *J. americana* tissues; and (H4) regardless of nutrient concentrations, *J. americana* tissue δ^15^N values would increase at mussel‐generated hotpots because more of the available N will be animal‐derived.

## 2 MATERIALS AND METHODS

### 2.1 White‐tailed deer sampling

**FIGURE 2:**
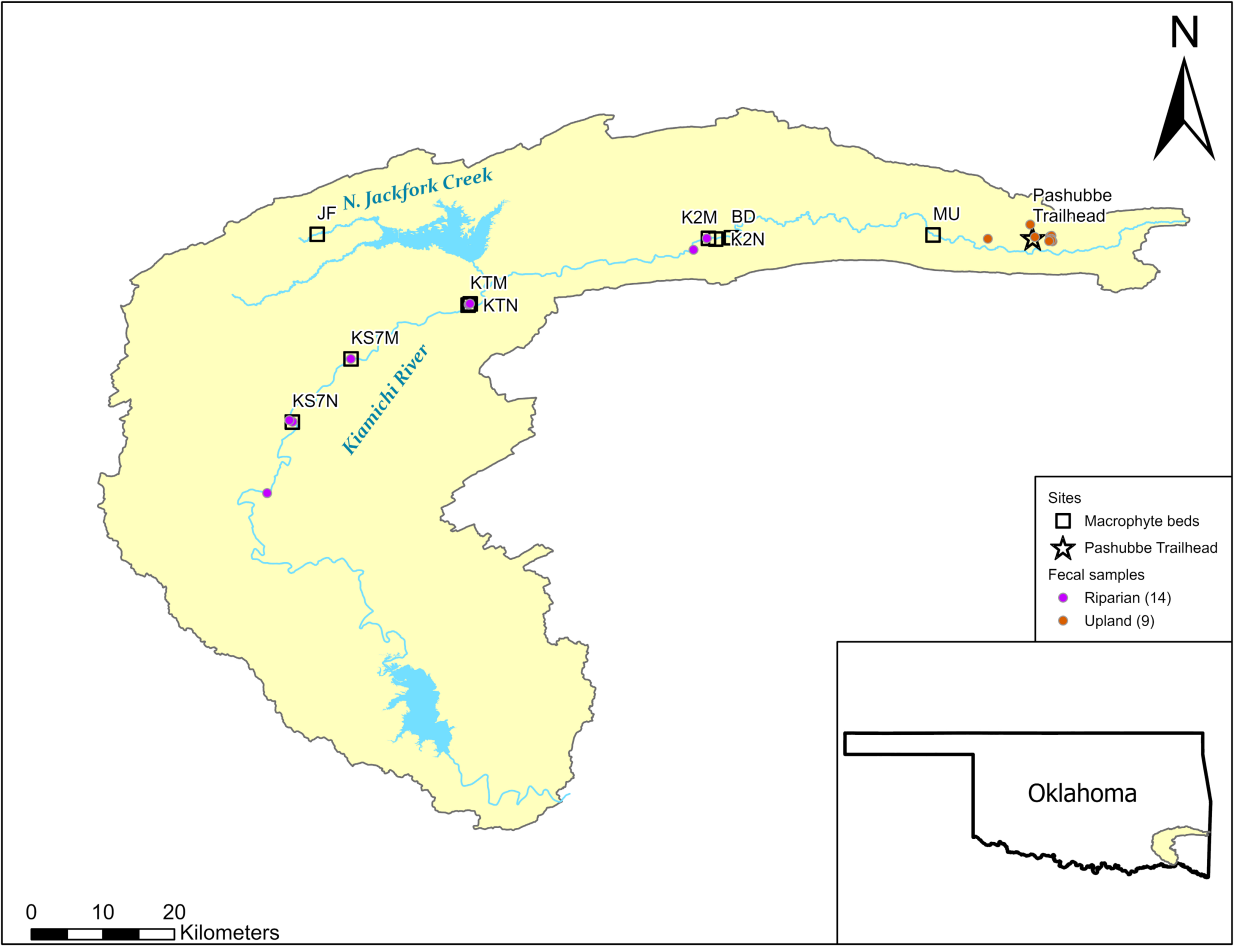
Map of the locations of white‐tailed deer fecal samples from riparian and upland locations, and macrophyte beds sampled within the Kiamichi River watershed. Inset shows the Kiamichi watershed within the US state of Oklahoma.

All sampling for the study described herein was conducted in the Kiamichi River watershed of southeastern Oklahoma, USA (Figure 2). To test for differences in diet between deer in riparian and upland habitats, we collected 23 deer fecal samples from game trails surrounding feeding areas from July 26–August 1, 2019, and June 16–August 4, 2021. We compared 14 samples collected from trails and gravel bars along the Kiamichi River (riparian samples) to 9 samples collected from trails running to and from wildlife clearings in the Ouachita National Forest along Pashubbe Trailhead (upland samples; Figure 2). Riparian samples represent fecal samples collected from the middle reaches of the Kiamichi River, where macrophyte and mussel beds are abundant. Upland samples represent fecal samples from near high‐gradient tributaries with no large macrophyte beds or mussels. Fecal samples retain nutrients for at least 24 days under normal environmental exposure (Jenks et al., 1990). Samples beyond 24 days old experience cracking and erosion (Jenks et al., 1990), so samples with these characteristics were not collected.

We quantified carbon (C) and nitrogen (N) content and isotopes in fecal pellets using a Thermo Isolink CN Elemental Analyzer integrated with a Thermo Delta V Advantage IRMS through a Conflo IV (Thermo Fischer Scientific). δ^13^C and δ^15^N values were calibrated using externally certified standards (USGS 40 and 41a for δ^15^N relative to air and δ^13^C relative to VPDB, and an algae (*Spirulina*) standard (Elemental Microanalysis Limited) for C and N content). The algae (*Spirulina*) standard was used for QA/QC and had an average standard deviation of <0.2‰ for both δ^13^C and δ^15^N between sample runs. Total phosphorus (P) content was estimated by combustion at 500°C and acid digestion at 105°C followed by soluble reactive phosphorus (SRP) analysis by the molybdate blue method (U.S. Environmental Protection Agency, 1983). We also analyzed the isotopic and nutrient composition of 10 greenbrier (*Smilax* spp.) leaf samples collected at each upland fecal sample location. *Smilax* spp. is a preferred food source of deer in the Ouachita Forest (Segelquist & Pennington, 1968), and one of the dominant understory vegetation taxa. Because terrestrial plants tend to be relatively restricted in their isotopic composition (Finlay & Kendall, 2007), and because *J. americana* is by far the dominant macrophyte we have observed in the study river, comparisons of the isotopic values between *Smilax* spp. and *J. americana* are expected to be broadly representative of the terrestrial and aquatic feeding options available to deer in the watershed. We compared the isotopic and nutrient composition of *Smilax* spp. and *J. americana* leaves to upland and riparian feces to assess the relative roles of terrestrial vegetation and macrophytes in the deer diet.

To test whether terrestrial herbivores more frequently consumed macrophytes at mussel‐generated hotspots, we analyzed data collected during a motion‐sensing game camera survey (Model# TR10i35A‐7; Wildgame Innovations) originally described anecdotally, but not analyzed, in Lopez et al. (2020). Briefly, we identified terrestrial vertebrate herbivores that visited *J. americana* beds, triggering a 30‐s time‐stamped video, and whether they were observed consuming *J. americana*. We placed cameras at 10 stream reaches, but flooding caused the loss of five cameras. One additional camera malfunctioned and ceased recording data early in the survey, leaving us with only four stream reaches at which we could compare herbivore activity; two cameras overlooking stream reaches that contained mussel‐generated biogeochemical hotspots and two reaches with no mussels. The loss of equipment limited our ability to compare deer behavior between locations. However, we still explored whether the data we were able to retrieve aligned with our hypotheses by comparing differences in the frequencies with which white‐tailed deer visited and foraged at mussel reaches (sites KTM and KS7M) and non‐mussel reaches (sites K2N and KTN). We also compared the proportion of individuals counted at each site that were seen eating *J. americana*.

### 2.2 Nutrient and macrophyte sampling

To explore whether mussel‐generated hotspots were associated with variability in nutrient availability and macrophyte density, we sampled nine gravel bars with nearly monoculture *J. americana* beds along a natural mussel density gradient from July 10 to August 14, 2019. Eight sites were along an ~118 km stretch of the Kiamichi River OK, and one site was on North Jackfork Creek—a major tributary of the river (Figure 2). Four sites contained no mussels, and five contained mussel beds of varying densities (~3–38 ind m^−2^). To test the potential effect of mussels on ambient nutrients, we estimated gravel bar porewater nutrient concentrations within *J. americana* beds at each site. We sampled ammonium (${\mathrm{NH}}_{4}^{+}$‐N) by the phenol‐hypochlorite method and SRP using the molybdate blue method (U.S. Environmental Protection Agency, 1983). We chose these forms because they are highly bioavailable and are the forms of N and P excreted by mussels. We took a composite porewater sample from the upstream and the downstream end of the macrophyte bed at each site. The samples were too high in sediment to filter in the field and were frozen until analysis because we had no capacity to analyze nutrient concentrations at our remote field sites. Freezing of porewater may cause minor losses of ${\mathrm{NH}}_{4}^{+}$‐N, so these data should be treated as conservative estimates (Yorks & McHale, 2000). Upon return to the laboratory, we thawed and decanted these samples into a syringe filter and filtered them using GF/F filters (0.7 μm).

To test for potential effects of mussels on *J. americana* density and tissue nutrient and isotope composition, we established 0.25 m^2^ plots across a representative spatial distribution of the *J. americana* bed at each site and sampled macrophyte density and nutrient composition within them. We determined plot density by measuring the length of each *J. americana* bed parallel to the direction of stream flow (range = 13–113.4 m) and sampled a minimum of one plot per 15 m (range = 2–10 plots). We also sampled environmental covariates that can influence plant growth and nutrient composition: light availability (percent shade using a spherical densiometer), and the median sediment grain size by measuring the length of the medial axis of 50 individual grains (Wolman, 1954). At each plot, we counted the total density of *J. americana* stems, as well as the proportion of broken or clipped stems, as physical damage may stimulate compensatory growth or nutrient responses in plants (McNaughton, 1983). We used density rather than biomass as an indicator of *J. americana* growth because herbivory and nutrient enrichment tend to have counteracting effects of similar magnitude on producer biomass (Gruner et al., 2008), although we did harvest aboveground *J. americana* tissue in each plot for nutrient analyses. Aboveground tissues were separated into leaves and stems and dried at 70°C for 72 h then ground in a knife mill. We assessed C:N:P stoichiometry in leaves and stems using molar ratios and δ^13^C and δ^15^N isotopic signatures via the methods described above for fecal samples.

### 2.3 Data analysis

We conducted all analyses in R v4.1.2 (R Development Core Team, 2021). We used Wilcoxon rank‐sum tests to compare the counts of white‐tailed deer observed at the paired sites with motion‐sensing cameras, the frequency with which they were seen feeding on *J. americana*, and the stoichiometry of deer fecal samples between upland and riparian habitats. Mean (±*SE*) values for site‐level *J. americana* and upland *Smilax* spp. leaf tissue and deer fecal pellet stoichiometry and isotopes are reported in Appendix S1: Table S1. We also plotted the isotopic composition of the fecal samples in relation to *Smilax* spp. and *J. americana* to assess whether *J. americana* contributes more to the diet of deer in riparian habitats than deer in upland habitats. We chose not to use a mixing model to test this hypothesis because we could not reasonably assume the two food resources that we sampled comprise the entire diet of deer (Phillips et al., 2014). Instead, we used a PERMANOVA (package vegan; Oksanen, 2022) to test whether the isotopic composition of riparian and upland deer fecal pellets was significantly different.

We averaged all nutrient and macrophyte data at the site level to test our hypotheses across all study sites (Appendix S1: Table S2). We used linear models to assess the relationships of *J. americana* stem density, nutrient stoichiometry, and isotopic composition to mussel density and the biotic and abiotic covariates described below. To determine what variables were related to porewater nutrient concentrations, as well as *J. americana* density and stoichiometry, we used ordinary least squares regression due to the method's flexibility to include multiple predictors. We selected the best subset of potential drivers using a regression model selection approach. We selected from mussel density and median sediment grain size as potential drivers in the models of porewater nutrient concentrations. We selected from percent shade (light effects), proportion of damaged or clipped stems (compensatory effects), sediment size (physical effects) and porewater ${\mathrm{NH}}_{4}^{+}$‐N:SRP ratio (nutrient effects) as potential drivers of *J. americana* density and stoichiometry. Because mussel presence is often correlated with sediment stability (Lopez & Vaughn, 2021), we also tested for a correlation between mussels and sediment size using a Spearman correlation test. We selected the best model based on differences in Akaike Information Criterion with correction for small sample size (ΔAIC_c_). Due to the large number of models tested, when multiple models for a given response variable had ΔAIC_c_ values <2 (indicating similar fit), we present only the model that explained the most variance based on its adjusted *R* ^2^ value. To test *J. americana* δ^15^N response to mussel density, we only had one driver to consider, so we used Seigel's robust regression (package *mblm*; Komsta, 2013) to decrease the influence of high‐leverage points in this small dataset. The parameters and statistics describing each model are reported in Appendix S1: Table S3.

## 3 RESULTS

### 3.1 *Justicia americana* is nutrient‐rich and aligns with riparian white‐tailed deer fecal isotopes

**FIGURE 3:**
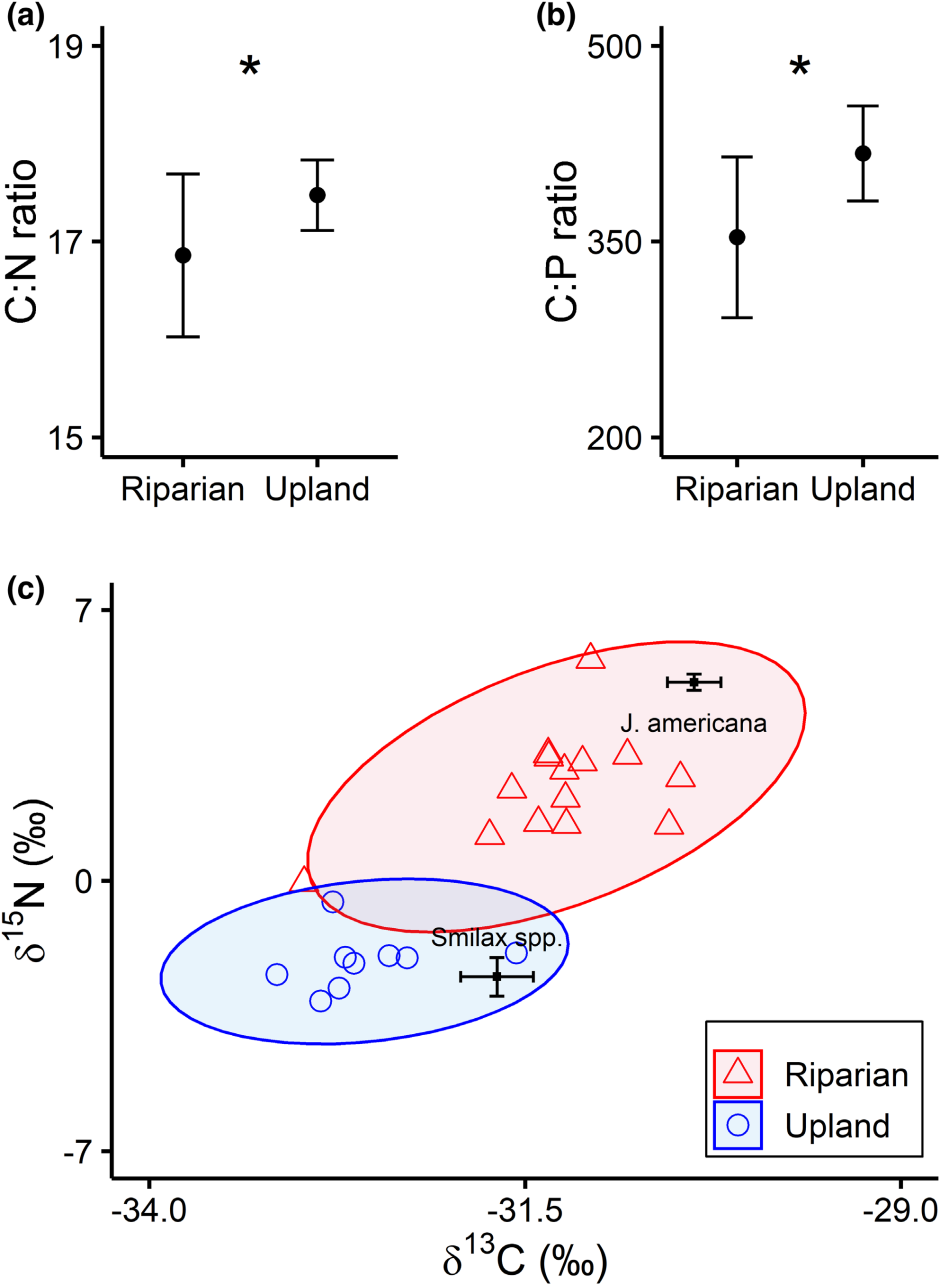
Comparison of the stoichiometric and isotopic composition of *Odocoileus virginianus* fecal samples from riparian and upland habitats. Riparian samples had significantly lower (a) C:N (*p* = .033) and (b) C:P ratios (*p* = .009) than upland samples, indicating a more nutrient‐rich diet. (c) Biplot comparing isotopic signatures of two deer food items—*Justicia americana* (aquatic) and *Smilax* spp. (terrestrial)—with deer fecal samples. Black points with error bars show mean (±*SD*) values of food sources. Colored points show fecal samples with 95% CI ellipses. Deer diets in the riparian habitats were significantly different from upland habitats (*p* = .001).

Deer fecal samples collected from the Kiamichi River riparian zone had 3% lower C:N (*W* = 29, *p* = .033; Figure 3a) and 20% lower C:P ratios (*W* = 22, *p* = .009; Figure 3b) on average than fecal samples from the Kiamichi Valley uplands, consistent with higher diet quality in deer accessing the riparian zone. Riparian samples also had 26% higher N:P ratios (*W* = 29, *p* = .035), indicating the egestion of more excess N relative to P. Riparian and upland fecal samples differed in isotopic composition (*F*_1,21_ = 73.25, *R* ^2^ = .78, *p* = .001; Figure 3c). Upland fecal samples clustered close to *Smilax* spp. but were depleted in ^13^C compared with *Smilax* spp. indicating that we must be missing additional dietary sources for upland deer—a limitation of our study design. However, riparian fecal samples clustered between *Smilax* spp. and *J. americana* in isotopic space, indicating higher prevalence of aquatic vegetation in the riparian deer diet.

### 3.2 Terrestrial herbivores fed on macrophytes more frequently at mussel‐generated hotspots

**FIGURE 4:**
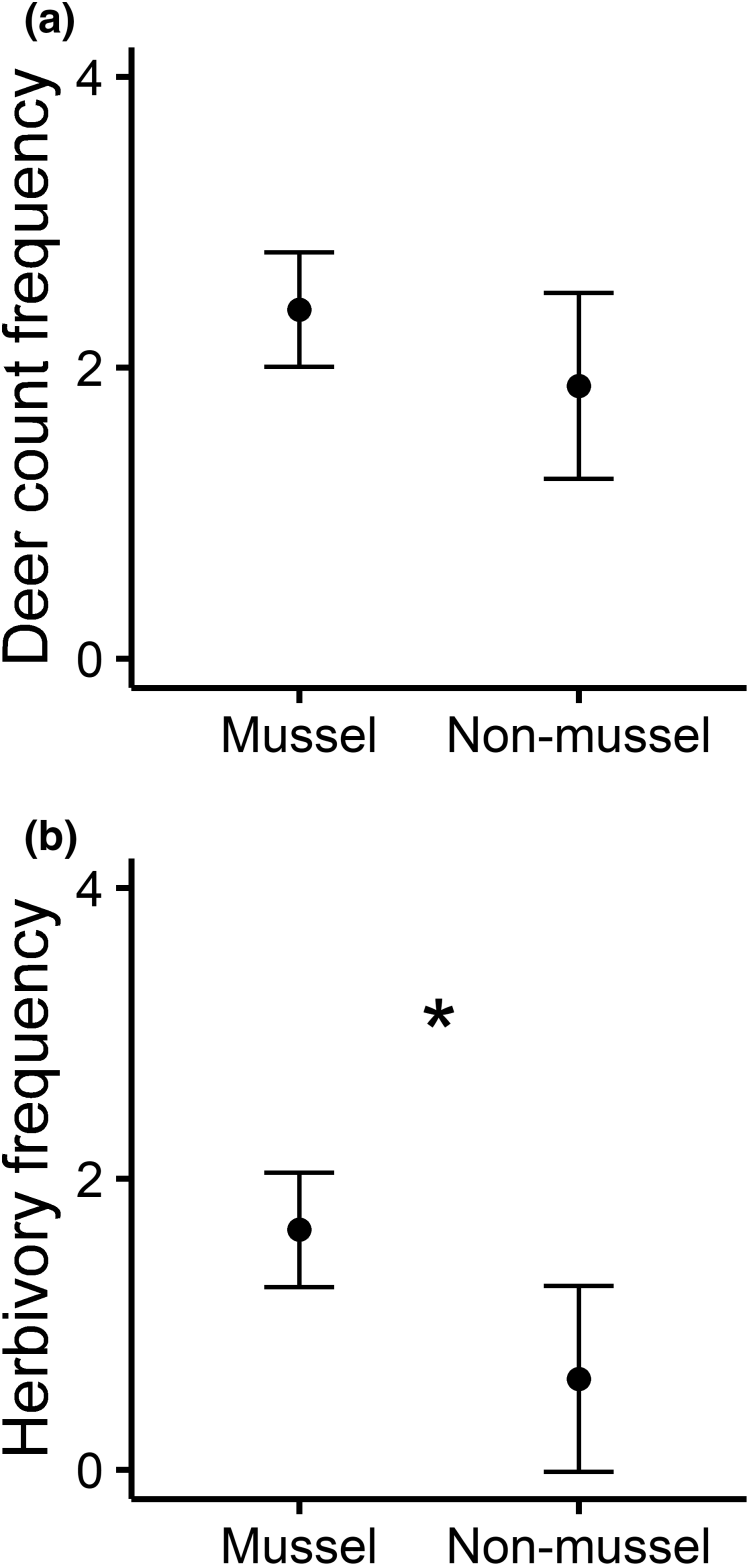
Counts of *Odocoileus virginianus* behavior at *Justicia americana* beds (per video recorded). (a) *Odocoileus virginianus* visited mussel sites at a similar rate as non‐mussel sites (*p* = .228). (b) *Odocoileus virginianus* consumed *J. americana* more frequently mussel sites than non‐mussel sites (*p* = .039).

The number of deer counted per video was similar between mussel (2.40 ± 0.39) and non‐mussel (1.88 ± 0.64) sites (*W* = 102.5, *p* = .228; Figure 4a). However, frequency of herbivory events on *J. americana* at mussel sites (1.65 ± 0.39) was over 2.5 times higher than at non‐mussel (0.63 ± 0.38) sites (*W* = 0.69, *p* < .039; Figure 4b). When analyzed proportionally, herbivory on *J. americana* was marginally more common among individuals seen at mussel sites (64 ± 8%) than at non‐mussel (31 ± 16%) sites (*W* = 113, *p* = .085).

### 3.3 Mussel‐generated hotspots are not strongly associated with *J. americana* bed macronutrients

**FIGURE 5:**
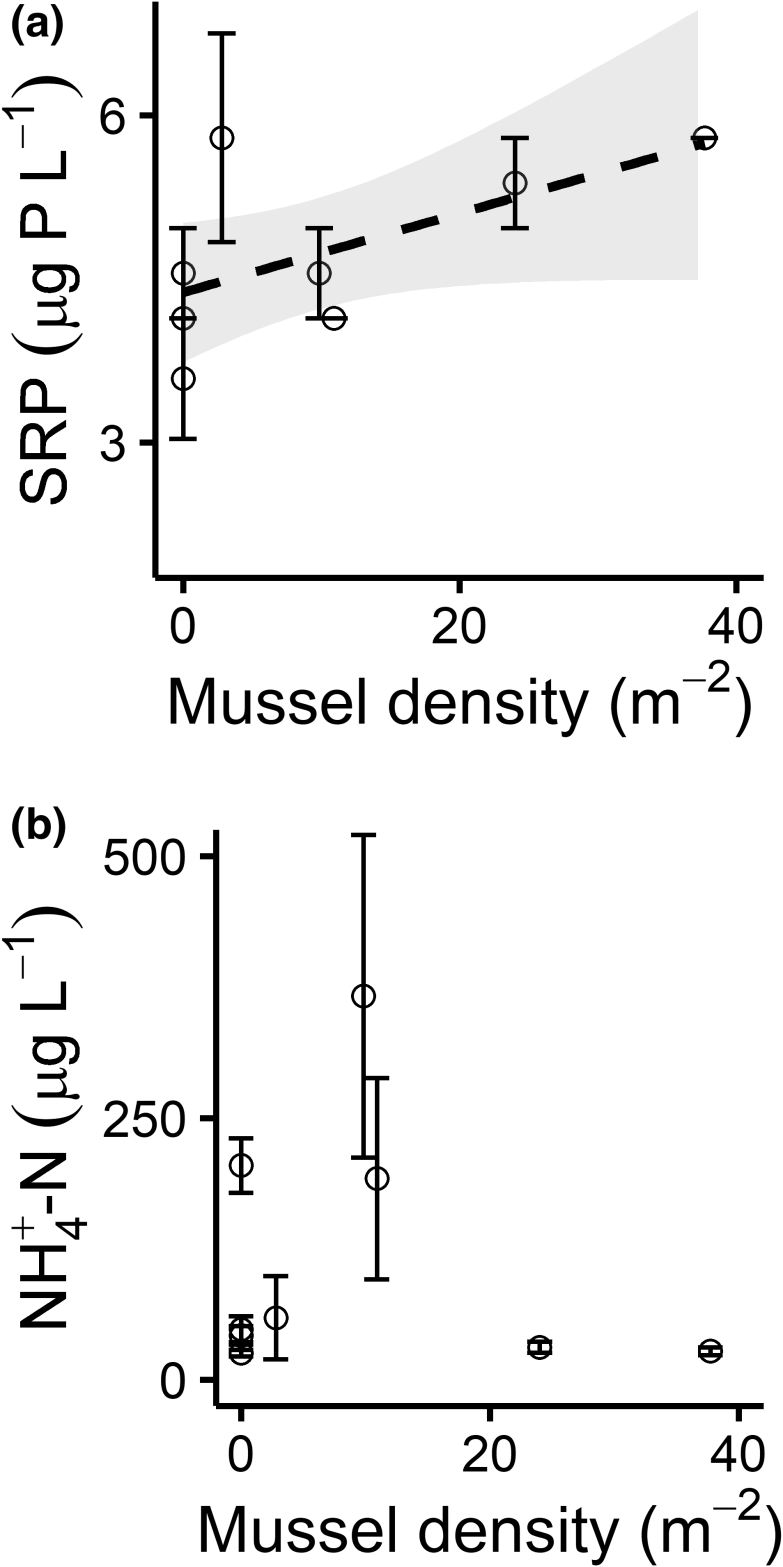
(a) Increased freshwater mussel density was associated with marginally increased porewater SRP concentrations (*y* = 0.04*x* + 4.38, *p* = .073). (b) Porewater NH_4_^+^‐N concentrations did not change in association with mussel density after accounting for sediment grain size (*p* = .580).

Mussel density was not strongly related to macronutrient concentrations in *J. americana* beds, although porewater SRP (soluble reactive phosphorus) did slightly increase in association with mussel density (Figure 5a). SRP increased by 62% across the mussel density gradient (*β* = 0.04, model: *F*_1,7_ = 4.44, *p* = .073, *R* ^2^ = .30). Porewater ${\mathrm{NH}}_{4}^{+}$‐N did not strongly covary with mussel density or sediment size (Figure 5b). There was also no relationship between median sediment grain size and mussel density (*p* = .580, *ρ* = 0.21). *J. americana* stem density did vary with porewater nutrient availability, but the effect did not appear to be associated with mussel density (Appendix S1: Table S3). Rather, the sixfold variation in stem density detected among sites was positively related to porewater ${\mathrm{NH}}_{4}^{+}$‐N:SRP ratio (*β*_1_ = 1.24, partial *R* ^2^ = .56) and was constrained by the negative effect of percent shade (*β*_2_ = −4.75, partial *R* ^2^ = .58), suggesting potential co‐limitation of *J. americana* growth by light and N (model: *F*_2,6_ = 10.86, *p* = .010, *R* ^2^ = .71).

*Justicia americana* tissue stoichiometry did not respond to porewater nutrient stoichiometry, further indicating a lack of any mussel‐related macronutrient effect on *J. americana*. Increasing median sediment size and light availability tended to increase C content. Leaf C:P varied by 65% across sites, increasing with sediment size (*β*_1_ = 2.67, partial *R* ^2^ = .63) but decreasing with percent shade (*β*_2_ = −3.34, partial *R* ^2^ = .67), suggesting that leaf C content is associated with light and physical habitat structure at our sites (model: *F*_2,6¸_ = 8.68, *p* = .017, *R* ^2^ = .66). Increases in sediment size were also associated with increases of 42% in stem C:N (*β* = .26, model: *F*_1,7_ = 11.34, *p* = .012, *R* ^2^ = .56). No other associations between *J. americana* tissue stoichiometry and the drivers we tested were detected (Appendix S1: Table S3).

**FIGURE 6:**
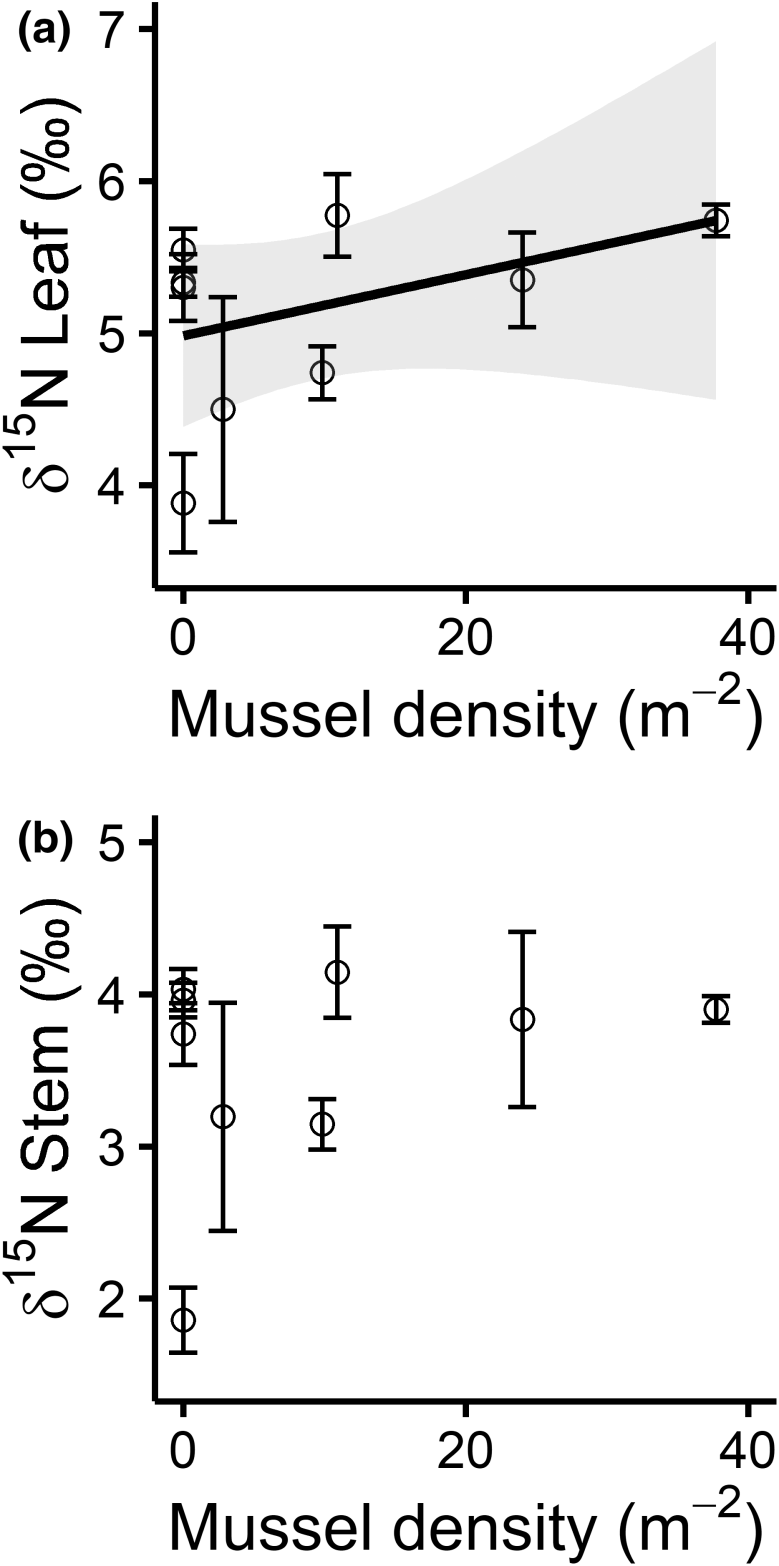
(a) δ^15^N values in *Justicia americana* leaf tissues increased with mussel density (*y* = 0.02*x* + 4.98, *p* = .020), likely indicating an increase in animal‐derived N being assimilated. (b) δ^15^N values in *J. americana* stem tissues did not change in association with mussel density (*p* = .164).

When the isotopic composition of *J. americana* aboveground tissue was analyzed, we found that increasing mussel density corresponded to a 49% enrichment in δ^15^N in *J. americana* leaf tissues (*β*_1_ = 0.02, *V*_7_ = 42, *p* = .020; Figure 6a), but that stem δ^15^N appeared to be unrelated to mussel density (*V*_7_ = 35, *p* = .164; Figure 6b).

## 4 DISCUSSION

This study provides evidence that white‐tailed deer are a previously unrecognized vector for aquatic‐derived nutrients to flow into nearby terrestrial ecosystems via herbivory on emergent macrophytes and subsequent defecation on land. White‐tailed deer feces in terrestrial riparian habitats were more nutrient rich and showed isotopic signatures closer to aquatic macrophytes than feces in upland habitats, which aligned closer to terrestrial vegetation (supporting H1 and H2). Although our motion‐sensing camera survey was limited by flooding, we did find that white‐tailed deer fed more frequently on macrophytes at freshwater mussel‐generated biogeochemical hotspots. This pattern aligned with H2, but due to the loss of equipment and resultant small sample size, further evidence is needed to claim support or lack thereof for this hypothesis. N and P dynamics did not covary with mussel density as we initially hypothesized (contrary to H2 and H3). However, when N isotopes in *J. americana* leaves were compared across a mussel density gradient, our findings were consistent with the notion that greater amounts of animal‐derived N were present at sites with mussel‐generated hotspots, likely because of N excretion by mussels (supporting H4).

As we hypothesized in H1, deer fecal C:N and C:P ratios were significantly lower in riparian fecal samples, consistent with higher diet quality (Leslie et al., 2008)—although the magnitude of the effect was relatively small for C:N. Alternatively, one might expect microbial and fungal mineralization to influence nutrient content depending on sample age or the surrounding habitat. However, N mineralization of cervid feces is relatively consistent between riparian and upland habitats, and N content is stable across 24+ days of exposure (Guernsey et al., 2015; Jenks et al., 1990), so we find it likely that differences were related to diet. Isotopic differences in deer fecal samples between upland and riparian habitats partially supported our second hypothesis H2, as riparian samples were more closely aligned with signatures of macrophytes, and upland fecal samples aligned very close to the signature of *Smilax* spp. These two separate clusters are consistent with known differences in the composition of terrestrial and aquatic plants, with riparian samples and macrophytes tissues both being enriched in ^15^N and ^13^C relative to upland samples and terrestrial plants (Finlay & Kendall, 2007; France, 1995; Milligan & Humphries, 2010). Thus, we suspect that macrophyte consumption was consistently greater in white‐tailed deer in riparian zones than upland habitats in the Kiamichi watershed. Aquatic macrophytes are generally higher in nutrients and lower in C than terrestrial plants (Bakker, Wood, et al., 2016); this held true in our study when comparing *Smilax* spp. and *J. americana*. Thus, we suggest that deer prefer macrophytes in their diet when they are available and may seek them out because they are richer in nutrients than terrestrial plants.

We also found potential evidence in support of H2 in our motion‐sensing camera survey, as deer did feed on *J. americana* more frequently at mussel‐generated hotspot sites than non‐mussel sites—albeit in a strongly limited sample. However, we found no evidence for the influence of mussel aggregations on macrophyte bed N and P dynamics. Due to these limitations, we cannot claim with conviction that deer prefer to feed on *J. americana* at freshwater mussel beds. However, we can speculate on reasons why such a spatial pattern in deer herbivory may have been observed in the data we were able to retrieve. Such explanations range from the accessibility of a given site, to the amount of cover from predators, to the surrounding land use. Although the macrophyte beds we studied are essentially monocultures, the surrounding riparian habitat can vary. For example, 8–15.4% of the land in the Kiamichi watershed is used for agricultural purposes, but much of this agricultural land use occurs along the mainstem of the river (Atkinson et al., 2012; Atkinson, Julian, & Vaughn, 2014). Most of this agricultural land is occupied by pasture for cattle farming (USDA National Agricultural Statistics Service, 2017), which may induce competition between deer and livestock, driving deer to exploit alternative food sources (Jenks et al., 1996). If the mussel sites that we surveyed happened to be surrounded by more cattle farms than the non‐mussel sites, competition might drive deer to exploit macrophytes more frequently as a food source. While we cannot rule out such alternative explanations, we also cannot rule out that mussel‐generated hotspots may contribute to preferential feeding by deer. Micronutrients tend to be richer in aquatic than terrestrial vegetation and may play a role in driving cervids to feed on macrophytes (Ceacero et al., 2014; Fraser et al., 1984). In a prior study of micronutrient availability conducted at the same sites, higher mussel density was correlated with increases in *J. americana* calcium, iron, copper, and zinc content (Lopez, 2022). Micronutrient demand—especially for calcium—is critical to antler formation in male cervids and to the reproductive needs of females, and deer are thought to actively seek out calcium‐rich foods (Jones & Hanson, 1985; Moen & Pastor, 1998). If the pattern of deer eating macrophytes more frequently at mussel‐generated hotspots is indeed representative of a broader scale process, it may be driven by elevated concentrations of micronutrients rather than for the macronutrients we studied in the present investigation. However, the present study does not contain sufficient data to confirm such a conclusion.

Contrary to our third hypothesis H3, mussel‐generated biogeochemical hotspots did not have strong effects on macrophyte bed N and P dynamics, and thus mussel‐driven macronutrient effects do not appear related to deer‐vectored resource fluxes. SRP in gravel bar porewater only marginally increased with mussel density and our models did not explain variation in ${\mathrm{NH}}_{4}^{+}$‐N concentrations well. ${\mathrm{NH}}_{4}^{+}$‐N concentrations varied over a much larger range than mussels have been shown to affect (Trentman et al., 2018), so ${\mathrm{NH}}_{4}^{+}$‐N dynamics within our study gravel bars are likely controlled by a combination of physical and microbial processes (Zarnetske et al., 2011). Macrophyte density increased significantly with porewater ${\mathrm{NH}}_{4}^{+}$‐N:SRP ratio, aligning with previous work demonstrating that algal production in the Kiamichi River is N‐limited (Atkinson et al., 2013; Vaughn et al., 2007). However, we also found that porewater ${\mathrm{NH}}_{4}^{+}$‐N:SRP exceeded the N:P of *J. americana* tissues in most cases—a pattern more consistent with P limitation. Experimental nutrient additions would be needed to determine whether *J. americana* growth is truly N‐limited in this system. Macrophyte C:N:P stoichiometry responded to environmental factors in a few cases but was mostly invariant between sites. This also contradicted hypothesis H3, but aligns with ecological syntheses that suggest plants are more homeostatic in their nutrient composition that previously thought (Borer et al., 2013; Demars & Edwards, 2007; Elser et al., 2010).

Yet, we did still find some support for hypothesis H4, as *J. americana* leaves became enriched in δ^15^N as mussel aggregations became denser. This indicates that *J. americana* was likely assimilating animal‐derived N (Erskine et al., 1998), which, given the comparative nature of this study, is presumably from mussels. Alternatively, we also considered that variability in the size and proximity of nearby cattle farms might impact ^15^N signatures in our study. Inorganic fertilizers are known to alter biotic ^15^N signatures in streams; however, animal manure does not have the same strong effects—much of the N it contains tends to release to the atmosphere through volatilization rather than leaching into runoff (Diebel & Zanden, 2009). Because agriculture in the Kiamichi watershed features little cropland that would require inorganic fertilizer application (USDA National Agricultural Statistics Service, 2017), we find it unlikely that runoff from nearby pastures impacted our isotope data. Stream‐dwelling animals such as mussels, on the contrary, release soluble ammonia directly into the water column (Spooner & Vaughn, 2008). Because macrophyte N isotopic composition is mainly a function of the nutrient's source rather than hydrologic or temporal variability (Chang et al., 2009; Pastor et al., 2014), we find it likely that in‐stream variability in ^15^N—associated with increasing mussel density—was responsible for the enrichment of *J. americana* leaf tissues. However, ^15^N enrichment was not reflected in stems. We suspect this can be explained by the fact that the N content of *J. americana* leaves was much higher than stems. Because leaves incorporated more ^15^N from the environment, it is reasonable to assume that effect sizes of δ^15^N enrichment in the smaller stem N pool could be too small to be detected across the present mussel density gradient. This assumption aligns with prior work showing that mussels increased *J. americana* leaf and stem δ^15^N enrichment in the presence of a steeper mussel density gradient (Lopez et al., 2020).

We have demonstrated that white‐tailed deer can be a vector for aquatic‐to‐terrestrial resource flows. We have presented direct behavioral observations that demonstrate that deer eat emergent macrophytes in the Kiamichi watershed. These behavioral observations are complemented by a comparative analysis of the isotopic composition of deer food and feces from contrasting aquatic and terrestrial habitats that supports the idea that deer deposit feces enriched in macrophyte‐derived nutrients into the terrestrial part of the riparian zone. The deposition of nutrients from aquatic source ecosystems should have functional implications for the recipient terrestrial ecosystems, as resource flows in terrestrial habitats tend to be less concentrated than aquatic ones (Schindler & Smits, 2017; Shurin et al., 2006; Subalusky & Post, 2019). However, without assessing terrestrial ecosystem responses such as primary productivity, the degree to which deer‐mediated transfers of macrophyte‐derived resources represent a true “subsidy” is unknown. Stable isotopes also indicated that these resource flows likely include N that was derived from mussel excreta, suggesting that macrophytes may indeed provide a connection point between aquatic and terrestrial animals. However, the evidence that aquatic biogeochemical hotpots generated by mussels might strengthen this aquatic–terrestrial linkage was equivocal, and macronutrient dynamics were insufficient to explain the patterns in herbivory frequency that we observed using motion‐sensing cameras. This lack of mussel‐derived macronutrient effects indicates a lack of support for the idea that mussel‐generated hotspots have any impact on deer‐mediated aquatic‐to‐terrestrial resource flows. Yet, we do suggest the preliminary evidence from our camera survey supports further investigation into the relationships between the taxa studied here (mussels, macrophytes, and cervids) because they are all globally distributed (Chambers et al., 2008; Graf & Cummings, 2007; Heywood, 2010). Ultimately, further assessment of the ecosystem‐level impacts of deer‐mediated nutrient fluxes is needed to determine how important such aquatic‐to‐terrestrial pathways are.

Animal‐driven resource subsidies are integral to understanding biogeochemical flows. Although animals have traditionally been thought of as negligible players in global elemental cycles, we now know that they have radiating effects on the entire ecosystem and significantly alter elemental dynamics at large spatial scales (Doughty et al., 2015; Schmitz et al., 2018). As demonstrated here, new ways in which animals impact biogeochemical flows and cycles continue to be explored. However, range contractions and loss of animal biomass have altered and threaten to irreversibly damage the resource flows that support ecosystems (Estes et al., 2011; Laliberte & Ripple, 2004). Comprehensive understanding of the roles that animals play in concentrating and translocating nutrient and mineral resources provides the opportunity for targeted conservation or restoration actions that may help preserve or repair the biogeochemical pillars of earth's ecosystems.

## AUTHOR CONTRIBUTIONS

**Jonathan W. Lopez:** Conceptualization (lead); data curation (lead); formal analysis (lead); funding acquisition (supporting); investigation (lead); methodology (lead); visualization (lead); writing – original draft (lead); writing – review and editing (equal). **Daniel C. Allen:** Data curation (supporting); methodology (supporting); resources (equal); writing – review and editing (equal). **Caryn C. Vaughn:** Data curation (supporting); funding acquisition (lead); investigation (supporting); methodology (supporting); resources (equal); visualization (supporting); writing – review and editing (equal).

## CONFLICT OF INTEREST

The authors declare that they have no conflicts of interest.

## Supporting information


Appendix S1

## Data Availability

The data used in this manuscript is available via Open Science Framework under the following identifier: https://doi.org/10.17605/OSF.IO/6H2ZY.
