# Supplementary material for: White‐tailed deer consumption of emergent macrophytes mediates aquatic‐to‐terrestrial nutrient flows
Source: Ecol Evol. 2022 Sep 12;12(9):e9257. doi: 10.1002/ece3.9257 (PMC9465632; doi:10.1002/ece3.9257)
Supplement: Supplementary file 1 — Appendix S1 [file ECE3-12-e9257-s001.docx]

# APPENDICES

## Appendix 1.

Supplemental data tables.

### Table S1.

Means (SE) stoichiometric and isotopic composition of leaf tissue from *Smilax spp.* (*n* = 10) and *J. americana* across sites (*n* = 9), and of *O. virginianus* riparian (*n* = 14) and upland (*n* = 9) fecal pellets.

| Sample type | C:N | C:P | N:P | δ^13^C (‰) | δ^15^N (‰) |
| --- | --- | --- | --- | --- | --- |
| *Smilax spp.* leaves | 26.88 (0.68) | 1212.57 (69.34) | 1212.57 (2.54) | -31.69 (0.24) | -2.48 (0.50) |
| *J. americana* leaves | 16.71 (0.94) | 421.51 (23.58) | 421.51 (0.36) | -30.37 (0.18) | 5.13 (0.21) |
| Riparian | 16.86 (0.83) | 353.33 (61.52) | 20.07 (2.03) | -31.29 (0.16) | 2.42 (0.36) |
| Upland | 17.46 (0.36) | 417.91 (36.49) | 23.94 (2.17) | -32.57 (0.15) | -2.08 (0.24) |

Table S2.

Means (± SE) of all response, predictor, and environmental variables for each site. Stoichiometric variables are presented as molar ratios. Median sediment grain size and percent shade have no associated SE because they were collected at the site level.

|  |  | Site | | | | | | | |
| --- | --- | --- | --- | --- | --- | --- | --- | --- | --- |
| Variable | JF | MU | BD | K2N | K2M | KTM | KTN | KS7M | KS7N |
| Mussel density (m^-2^) | 2.80 (1.20) | 0  (n/a) | 10.91 (5.67) | 0  (n/a) | 9.8 (1.61) | 37.7 (1.71) | 0  (n/a) | 24 (3.01) | 0  (n/a) |
| Porewater SRP (µg P L^-1^) | 5.79 (0.96) | 4.55 (0.41) | 4.14 (0) | 3.58 (0.55) | 4.55 (0.41) | 5.79 (0) | 4.14 (0) | 5.38 (0.41) | 4.55 (0.41) |
| Porewater NH_4_^+^-N (µg N L^-1^) | 59.22 (23.05) | 25.38 (2.21) | 192.12 (96.19) | 42.30 (8.86) | 366.51 (154.15) | 27.20 (3.61) | 48.21 (12.25) | 31.02 (5.36) | 204.61 (15.85) |
| Porewater NH_4_^+^-N:SRP | 17.57 (9.66) | 12.41 (0.86) | 102.69 (51.41) | 29.82 (n/a) | 172.40 (72.53) | 11.22 (1.54) | 25.77 (6.55) | 12.99 (2.30) | 102.67 (13.67) |
| Shade (%) | 42.12 | 36.92 | 1.3 | 1.82 | 9.1 | 4.94 | 8.32 | 0 | 5.98 |
| Median sediment grain size | 22.3 | 41.55 | 0 | 0 | 22.95 | 48.5 | 48.45 | 26.7 | 16.75 |
| Stem density | 94.00  (18.00) | 70.00  (11.72) | 369.60 (45.86) | 223.20  (55.99) | 352.00  (21.50) | 218.40  (46.11) | 234.40  (68.96) | 269.33  (61.92) | 490.00  (90.24) |
| Stems clipped (%) | 50.47 (3.10) | 44.45 (4.78) | 0.15 (0.10) | 3.50 (1.02) | 45.62 (4.95) | 22.23 (9.19) | 56.96 (8.82) | 24.88 (5.53) | 10.94 (5.24) |
| C:N_leaf_ | 14.68 (0.66) | 12.86 (0.80) | 16.91 (1.16) | 13.21 (0.52) | 17.65 (0.71) | 20.14 (1.40) | 20.58 (0.46) | 15.77 (1.39) | 18.59 (0.91) |
| C:N_stem_ | 43.98 (1.52) | 48.93 (2.61) | 45.08 (2.09) | 39.39 (2.15) | 50.28 (2.09) | 56.02 (2.55) | 54.42 (1.71) | 41.08 (4.85) | 41.80 (1.71) |
| C:P_leaf_ | 344.56 (71.78) | 346.15 (17.50) | 416.52 (31.49) | 355.55 (21.10) | 424.93 (16.88) | 568.31 (30.46) | 466.47 (29.69) | 429.44 (43.32) | 441.64 (20.71) |
| C:P_stem_ | 579.87 (11.73) | 516.67 (16.34) | 405.69 (30.55) | 448.82 (34.47) | 467.19 (20.67) | 689.13 (76.76) | 485.08 (28.35) | 445.45 (82.32) | 302.44 (38.38) |
| N:P_leaf_ | 12.29 (2.03) | 14.30 (0.54) | 12.99 (0.37) | 14.27 (0.95) | 12.75 (0.56) | 15.00 (0.60) | 11.95 (0.68) | 14.34 (0.53) | 12.61 (0.80) |
| N:P_stem_ | 6.97 (0.38) | 5.63 (0.26) | 4.78 (0.37) | 6.14 (0.74) | 4.93 (0.28) | 6.55 (0.81) | 4.70 (0.24) | 5.78 (0.92) | 3.83 (0.51) |
| δ^13^C_leaf_ (‰) | -31.05  (0.53) | -30.73 (0.18) | -29.66  (0.11) | -29.80 (0.16) | -30.37 (0.18) | -29.91 (0.22) | -30.15 (0.24) | -31.01 (0.20) | -30.80 (0.22) |
| δ ^13^C_stem_ (‰) | -31.07 (0.39) | -29.82 (0.25) | -29.81 (0.25) | -29.59 (0.39) | -29.37 (0.44) | -33.32 (2.83) | -37.54 (3.04) | -28.46 (0.56) | -29.47 (0.08) |
| δ^15^N_leaf_ (‰) | 4.50 (0.74) | 3.88 (0.32) | 5.78 (0.27) | 5.33 (0.10) | 4.74 (0.17) | 5.74 (0.10) | 5.55 (0.14) | 5.35 (0.31) | 5.30 (0.22) |
| δ^15^N_stem_ (‰) | 3.20 (0.75) | 1.86 (0.21) | 4.15 (0.30) | 3.96 (0.11) | 3.15 (0.17) | 3.90 (0.09) | 3.74 (0.20) | 3.84 (0.58) | 4.03 (0.14) |

Table S3.

Regression models and their coefficient and intercept estimates (± SE for *lm* models; ± MAD for *mblm* models). Bolded table rows describe statistically significant models (α = 0.05). *F* statistics describe full *lm* models, *V* statistics describe the slope of *mblm* models. *R^2^* values are not applicable to median-based (*mblm*) models.

| *Model type*: response ~ terms | Model coefficients (*β_i_)* & intercepts (*β_0_*) | Test statistic | *P* | *R^2^_adj_* |
| --- | --- | --- | --- | --- |
| *lm*: porewater_NH_4_^+^-N ~ *β_1_*sed_size + β0 | *Β_1_ =* -2.41 (2.27)  *β_0_ =* 171.82 (69.55) | *F_1,7_* = 1.13 | 0.323 | 0.02 |
| *lm*: porewater_SRP ~ *β_1_*mussel_density + β0 | *Β_1_ =* 0.04 (0.02)  *β_0_ =* 4.38 (0.27) | *F_1,7_* = 4.44 | 0.073 | 0.30 |
| *lm*: porewater_ NH_4_^+^-N: SRP ~ *β_1_*sed_size + β0 | *Β_1_ =* -1.31 (1.10)  *β_0_ =* 87.70 (33.92) | *F_1,7_* = 1.41 | 0.274 | 0.27 |
| *lm*: **stem_density ~ *β_1_*pore_N:P + *β_2_*%_shade + *β0*** | ***β_1_* = 1.24 (0.44)**  ***β_2_* = -4.75 (1.65)**  *β_0_* = 248.98 (42.62) | ***F_2,6_* = 10.86** | **0.010** | **0.71** |
| *lm*: leaf_C:N ~ *β_1_*%_ shade + β0 | *β_1_* = -0.08 (0.06)  *β_0_* = 17.72 (1.15) | *F_1,7_* = 1.92 | 0.208 | 0.10 |
| ***lm*: leaf_C:P ~ + *β_1_*sed_size + *β_2_*%_ shade + β0** | ***β_1_* = 2.67 (0.83)**  ***β_2_* = -3.34 (0.97)**  *β_0_* = 394.76 (25.14) | ***F_2,6_* = 8.68** | **0.017** | **0.66** |
| *lm*: leaf_N:P ~ *β_1_*pore_N:P + β0 | *β_1_* = -0.01 (0.01)  *β_0_* = 13.84 (0.49) | *F_1,7_* = 1.77 | 0.223 | 0.09 |
| ***lm*: stem_C:N ~ *β_1_*sed_size + β0** | ***β_1_* = 0.26 (0.08)**  ***β_0_* = 40.32 (2.32)** | ***F_1,7_* = 11.34** | **0.012** | **0.56** |
| *lm*: stem_C:P ~ *β_1_*sed_size + β0 | *β_1_* = 3.53 (1.80)  *β_0_* = 392.83 (55.05) | *F_1,7_* = 3.87 | 0.090 | 0.26 |
| *lm*: stem_N:P ~ *β_1_*porewater_N:P + β0 | *β_1_* = -0.01 (0.01)  *β_0_* = 6.04 (0.40) | *F_1,7_* = 2.32 | 0.084 | 0.28 |
| ***mblm*: leaf_δ^15^N ~ *β*mussel_density + β0** | ***β_1_* = 0.02 (0.03)**  *β_0_* = 4.98 (0.52) | ***V_7_* = 42** | **0.020** | - |
| *mblm*: stem_δ^15^N ~ *β*mussel_density + β0 | *β_1_ =* 0.00 (0.02)  *β_0_ =* 3.73 (0.31) | *V_7_* = 35 | 0.164 | - |
